# Prevalence and Determinants of Exclusive Breastfeeding Practice among Mothers of Children Aged 6–24 Months in Hail, Saudi Arabia

**DOI:** 10.1155/2021/2761213

**Published:** 2021-03-27

**Authors:** Mashail Basheir Alshammari, Hassan Kasim Haridi

**Affiliations:** ^1^Family & Community Medicine Joint Program, Hail, Saudi Arabia; ^2^The Designated Institutional Official (DIO) for Academic Affairs & Postgraduate Studies, Health Affairs, Najran, Saudi Arabia; ^3^Research Department, Health Affairs, Hail Region, Saudi Arabia

## Abstract

**Background:**

The WHO recommends that infants should be exclusively breastfed for the first six months of life to achieve optimal growth, development, and health. Nonadherence to exclusive breastfeeding (EBF) depends largely on the individual, sociocultural context, and institutional factors. The aim of this study is to estimate coverage and factors associated with adherence to EBF among mothers in the urban Hail region, Saudi Arabia.

**Methods:**

A cross-sectional study was carried out during February–June 2019 among 450 mothers of children aged 6–24 months attending immunization and well-baby clinics in 6 primary healthcare centers in Hail city. A pretested structured questionnaire was used to interview the consented participants.

**Results:**

The majority of mothers (72.9%) were aware of EBF; 24% reported initiation of breastfeeding within one hour after delivery; however, 71.1% did during the first 24 hours. The majority (76.8%) fed colostrum to their newborn; nevertheless, 50.1% had given a prelacteal feeding. Mothers who reported EBF practice were 50.7% (CI 45.9–55.4). The adjusted logistic regression analysis revealed that mother's awareness about EBF (aOR 3.03; 95% CI 1.78–5.18), antenatal care received at the governmental facility (aOR 2.63; 95% CI 1.28–5.41), breastfeeding a previous child (aOR 2.42; 95% CI 1.46–4.03), counseling received after delivery (aOR 2.47; 95% CI 1.34–4.53), and colostrum feeding given (aOR 4.24; 95% CI 2.31–7.77) were positively associated with EBF practice. On the other hand, mother's education (OR 0.39; 95% CI 0.15–0.99), higher family income (aOR 0.04; 95% CI 0.00–0.31), and practice prelacteal feeding (aOR 0.61; 95% CI 0.38–0.97) were negatively associated with EBF practice.

**Conclusion:**

EBF rate in urban Hail is still far below WHO recommendations. Efforts to strengthen mothers' counseling/support during antenatal care and immediately after delivery are needed to promote EBF practice, especially in the private sector.

## 1. Introduction

Breastfeeding is an investment in health, not just a lifestyle decision. It provides unmatched health benefits for babies and mothers. Infants who are breastfed have reduced risks of asthma, obesity, Type 1 diabetes, severe lower respiratory disease, acute otitis media, sudden infant death syndrome, gastrointestinal infections, and necrotizing enterocolitis for preterm infants [1, 2]. Studies have shown also an inverse relationship between exclusive breastfeeding (EBF) and infant mortality rates in developing countries [3], so that WHO described EBF as the single most effective intervention to improve the survival of children [4]. Women who breastfeed also have a reduced risk of high blood pressure, Type 2 diabetes, ovarian cancer, and breast cancer [1, 2].

The WHO and the United Nations Children's Fund (UNICEF) recommend that children be exclusively breastfed for the first 6 months of life—meaning no other foods or liquids are provided, including water [1]. However, the global picture falls short of these standards, as only about 40% of infants aged 0–6 months old are exclusively breastfed [5]. This is far below the widely accepted “universal coverage” target recommended by WHO/UNICEF that there should be 90% EBF in children less than 6 months in developing countries [6, 7].

The WHO in the Eastern Mediterranean region set a regional strategy on nutrition 2010–2019 that the percentage of women exclusively breastfeeding for the first 6 months increased by 50% [8]. The World Health Assembly (WHA) in 2012 set this target to be reached at the global level by 2025 [9].

There is insufficient data available on breastfeeding in Saudi Arabia. An earlier study in 2009 reported a declining trend of exclusive breastfeeding from 90% to 30% at the age of 3 months [10]. To estimate the EBF rate in Saudi Arabia, a systematic review published in 2014 found high variation among studies, which ranged from 0.8% to 43.9%, and clinched that the EBF rate could not be accurately determined due to the lack of clear definitions and the nature of study design [11]. Furthermore, the WHO does not report any breastfeeding data in the country profile because there are no national data on breastfeeding [12].

Although there are several studies identifying rates and factors influencing EBF, still there is a need to assess rates and understand the specific factors that impact the promotion of breastfeeding at the local level. This area of knowledge is recognized by the WHO as a gab and considered it a research priority [13]. Therefore, this study aimed to identify the prevalence and factors associated with the practice of EBF among mothers in Hail city, Saudi Arabia.

## 2. Materials and Methods

### 2.1. Setting

A cross-sectional study was carried out between February and June 2019 in Hail city, in the north of Saudi Arabia, among mothers taking their children to one of six governmental primary healthcare (PHC) centers. PHC centers were selected at random between 24 centers covering all neighborhoods of Hail city. Among other services provided by PHC centers, well-baby and vaccination services are principal services provided free of charge. Vaccination of children is mandatory in Saudi Arabia, with coverage rates of 96–98% for all vaccines in children aged one to two years [14]. Therefore, the selected mothers can be considered a representative sample.

### 2.2. Participants

The sample was selected using a two-stage sampling method. In the first stage; from the list of 24 PHC centers, 25% of centers (six centers) were selected systematically with the first one at random. In the second stage, mothers of children 6–24 months, who visited the selected PHC centers seeking vaccination or routine checkup of their children, were randomly selected and invited to undergo an interview. Mothers were eligible if they were aged 18 years or over, with no medical condition preventing them or their children from breastfeeding.

### 2.3. Sample Size

The sample size was determined assuming that 50% of mothers practice EBF breastfeeding for up to six months to maximize sample size, a 95% confidence level, and a 5% margin of error. The nonresponse rate was considered at 10%; therefore, the final sample size was calculated to comprise 440 mothers.

### 2.4. Data Collection

Preparing for conducting the study, the authors visited the assigned PHC centers and met the directors of those centers, introduced the study objectives, and showed the official letters of the regional health authority to facilitate the study conduction and the letter of ethical approval. All centers approached agreed to participate. Data were collected through face-to-face interviews with the eligible mothers at random during the study period. One female researcher carried out all interviews with mothers who agreed to participate and gave their consent. The interviews were carried out privately for about 15 minutes.

### 2.5. Data Collection Tool

The interviews were carried out, guided by a questionnaire prepared by the study authors. Inquiries included in the questionnaire were based on previous relevant literature: international [15, 16], Middle East [17, 18], and Saudi Arabia [11, 19–21]. Other items that authors considered important to address the aims of the study were included. The questionnaire consisted of 4 parts: (1) sociodemographic characteristics of the participant mother; (2) mother's medical and obstetrical history, care received during pregnancy, labor, and puerperium; (3) information about the child characteristics and details about breastfeeding practice; and (4) mother's awareness, knowledge of breastfeeding, and source of information.

The face validity and content validity of the questionnaire were reviewed by a panel of 4 experts (pediatrician, nutritionist, family medicine, and public health). A pilot study done on 20 eligible mothers (not included in the final sample) was carried out before commencing the study; accordingly, the questionnaire was revised and modified to its final form.

### 2.6. Ethics

The study protocol was approved by the Bioethical Committee of the General Directorate of Health Affairs, Hail region, Saudi Arabia, with the ethical approval number being 2019–17. Agreed participants signed the study consent form.

### 2.7. Statistical Analysis

Data was entered, cleaned, and analyzed using Epi Info 7 (CDC, Atlanta, Georgia, US). Data was summarized using proportions for categorical data and mean and standard deviation for continuous data. The relationship was determined using chi-square for categorical variables and *T*-test or ANOVA test for continuous variables or nonparametric tests as applicable if data were not normally distributed.

Multivariable analysis was carried out using logistic regression analysis to find out factors independently associated with EBF practice among mothers. Mothers who practiced EBF (no = 0; yes = 1) were tested with predictor variables being assumed to affect this practice. Variables of the final model were determined using a stepwise backward removal method, deleting variables with a *p* value above 0.25 in order to exclude the nonimportant variables from the model until the minimum adequate model was reached. Odds ratios (ORs) as well as their 95% confidence intervals (CIs) were calculated for the predictor variables in the analyses. All statistical tests were two-tailed, and differences were considered to be statistically significant at a *p* value ≤0.05.

## 3. Results

### 3.1. Mothers' Sociodemographic Characteristics

A total of 450 successful interviews out of 480 mothers asked to participate in the study, which yielded a response rate of 93.8%. The main characteristics of the sample are described in Table 1. In particular, the average age of participating mothers was 30.2 ± 7.48 years. About half (45.6%) attained a university degree or higher, while illiterate women constitute a small percent (5.8%). Working mothers accounted for 30% of the participants.

### 3.2. Obstetric History and Health Service-Related Factors

The majority (79.1%) of mothers were multigravida; 78.8% of them had more than one live child. The mean number of antenatal care visits received was 4 visits (4.0 ± 1.68) and 62.5% received postnatal care. The majority (77.7%) received breastfeeding counseling during pregnancy and 79.9% immediately after delivery. Nearly all had given birth at full term (94.4%) and delivered normally (93.8%) at public hospitals (90.7%). Most of them (88.1%) breastfed their previous children (Table 1).

### 3.3. Awareness and Sources of Knowledge about Breastfeeding

**Figure 1:**
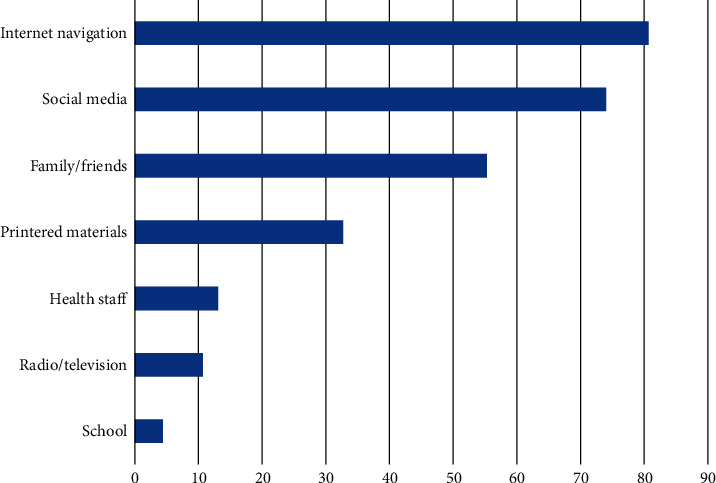
Frequency of use (%) of different sources of knowledge about breastfeeding among participants. Participants may report more than one source of knowledge.

Although 80.4% of mothers were aware of the concept of EBF and 72.9% of them correctly identified that exclusive breastfeeding means that the baby should receive only breast milk without any other supplements of any kind, only 21.6% of them correctly identified that the duration of EBF is for 6 months (Table 1). The main sources of the participants' knowledge about breastfeeding were Internet sites (80.7%), social media (74.0%), family and friends (55.3%), posters and pamphlets (32.7%), healthcare staff (13.1%), television and radio (10.7%), and school (4.4%) (Figure 1).

### 3.4. Breastfeeding Practice

The pattern of newborn feeding during the hospital stay and after discharge was explored among participants (Table 1). According to the participants' responses, only 24% of mothers reported that they put their newborn on breast within one hour after delivery; however, 71.1% did during the first 24 hours. About half of the participants (46.8%) initiated breastfeeding during their hospital stay, and 76.8% of them reported that they fed their newborn the colostrum; however, 50.1% had given their newborn a prelacteal feeding. The prevalence of EBF practice was 50.7% (95% CI 45.9, 55.4%). About 34.3% of the mothers were still breastfeeding their children during the study period.

### 3.5. Factors Associated with Exclusive Breastfeeding Practices

Results of the bivariate analysis to find the association between EBF and factors that might have influence are presented in Table 2. Factors that are found to be significantly associated with EBF and other important factors of interest are subsequently included in the multivariate logistic analysis to capture independent associations (Table 3). According to the multivariable logistic regression analysis, the following factors were positively associated with EBF practice: (i) mother's awareness about EBF (odds ratio (OR) 3.03; 95% confidence interval (CI) 1.78–5.18), (ii) antenatal care received at governmental healthcare setting (OR 2.63; 95% CI 1.28–5.41), (iii) history of breastfeeding of the previous child (OR 2.42; 95% CI 1.46–4.03), (iv) breastfeeding counseling received after delivery (OR 2.47; 95% CI 1.34–4.53), and (v) colostrum feeding given for the baby (OR 4.24; 95% CI 2.31–7.77). On the other hand, (i) mother's education (OR 0.39; 95% CI 0.15–0.99), (ii) higher family income (OR 0.04; 95% CI 0.00–0.31), and (3) practice of prelacteal feeding (OR 0.61; 95% CI 0.38–0.97) were negatively associated with EBF breastfeeding practice.

## 4. Discussion

Breastfeeding is an unequaled way of providing ideal food for the healthy growth and development of infants; it is also an integral part of the reproductive process with important implications for the health of mothers [1, 2, 22]. In 2012, the World Health Assembly (WHA) set a global target to increase the rate of EBF in the first 6 months up to at least 50% by 2025 [9]. The results of our study (50.7% EBF rate) indicate that this target has been achieved in the urban community of the Hail region, Saudi Arabia. However, it is still far away from the widely accepted “universal coverage” target recommended by WHO/UNICEF that there should be 90% EBF in children less than 6 months in developing countries [6, 7]. The prevalence of EBF in our study is higher than some other recent reports in Saudi Arabia as shown in Tabouk (31.4%; 2017) [19], Rabigh, at western region (27.6%; 2019) [23], and Taif (16.3%; 2019) [24] and as high as 37.0% (2018) in the capital Riyadh and Dammam main cities [21]. This wide variation in the rates of EBF was also reported in earlier regional reports in Saudi Arabia, which ranged from 0.8 to 43.9% [11]. The noticeable variation between studies indicates the importance of carrying out standardized national surveys covering all regions in the country to find out the national rate of EBF and to map the actual disparities between regions. National surveys should be conducted on a regular basis to observe the progress of the national strategies for breastfeeding promotion.

The relatively higher rate of EBF in our study challenges the conclusion that the EBF trend in Saudi Arabia is in decline [11], coping with the reported rising trend in developed countries [25].

Awareness about the concept of EBF among participants in our study was somewhat commendable (80.4%). Mothers who were aware of EBF were independently three times more likely to exclusively breastfed their children, irrespective of their education level. Similar findings have been reported by previous research [19] and indicate the importance of health education campaigns and other awareness programs to convey a clear message about the importance of EBF. Utilizing modern means of mass communication is of value since it is easily applicable and easily utilizable and will ensure a good diffusion of health education messages to a larger number of the target population and creates a positive norm toward breastfeeding in the community. Health information-seeking behavior among participants in our study indicated the heavy utilization of this means (80.7% navigated Internet sites and 74.0% of them used social media to get information about breastfeeding).

Even though it is a natural act, breastfeeding is also a learned behavior. Virtually, all mothers can breastfeed provided that they have accurate information and support within their families and communities and from the healthcare system [22]. Our results showed that mothers who received breastfeeding counseling immediately after delivery were two and half times more likely to exclusively breastfeed their children compared to those who did not receive such counseling. This confirms the findings of other studies [26, 27]. Breastfeeding counseling during antenatal care and immediately after delivery together with other elements of the baby-friendly hospitals [28] is important institutional practice universally followed in maternity hospitals and other maternity departments in general hospitals in the Hail region, applying the initiative of baby-friendly hospitals. However, adherence to these regulations is not tightly followed in private healthcare facilities, as revealed from the analysis of our study, where mothers who received antenatal care in governmental healthcare facilities were independently more than two and half times more likely to exclusively breastfed their children compared to those who received antenatal care in private facilities. This indicates that government healthcare facilities are more compliant with the guidelines of WHO/UNICEF and the Ministry of Health regarding breastfeeding promotion compared to private healthcare facilities. It implies also that the private healthcare facilities should be supervised well to comply with the initiative of baby-friendly hospitals and training of healthcare staff and tightly apply the code of marketing human milk substitutes.

There should be compliance with breastfeeding promotion guidelines regarding initiating breastfeeding early after delivery, giving colostrum to the newborn, and not giving any prelacteal feeds which were independent predictors of EBF practice among our study participants. Mothers who initiated breastfeeding early by giving colostrum to their newborns were more than 4 times more likely to exclusively breastfeed their children, and those who did not give a prelacteal feeding were one and half times independently more likely to exclusively breastfeed their children. This finding supports reports of other research studies [20]. These practices mostly occur after delivery while the mother is still in the hospital so that maternal counseling and support are two crucial approaches to promote EBF among mothers to be emphasized.

**Figure 2:**
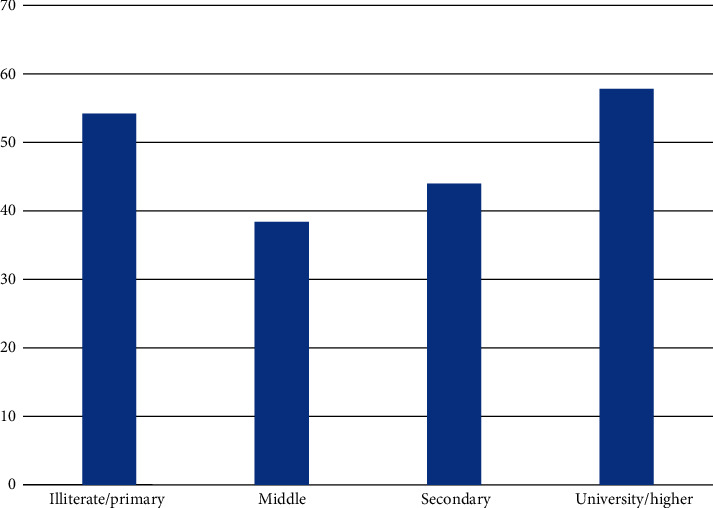
Prevalence of exclusive breastfeeding practice among mothers at education levels.

In our analysis, we found that educated mothers were independently more likely to discontinue EBF compared to illiterate ones. Educated mothers in general were 60% less likely to exclusively breastfed their children as revealed by logistic regression analysis compared to illiterate ones. This finding is also reported in some other research studies from Saudi Arabia and developing countries [11, 20, 29–32]. However, education in the bivariate analysis in our study showed a U-shaped association (Figure 2), where mothers who were illiterate or just have primary schooling (EBF 54.2%) and those with university or higher level of education (EBF 57.8%) were more adherents to EBF, compared to mothers with middle (EBF 38.3%) or secondary schooling (EBF 44.0%). A possible explanation for these findings for the illiterate/low educated mothers is the more intimacy to the traditional life, where breastfeeding is seen as the main role and the responsibility of motherhood and is a translation of what was seen and practiced by their mothers. Mothers with university or higher education might potentially have higher breastfeeding literacy and be convinced of the importance of breastfeeding for child and mother's health as seen in developed countries [33–36]. The U-shaped effect of a mother's education on adherence to breastfeeding might explain in part the conflicting results of studies that reported a positive association and those that reported a negative one.

Poorer mothers in our study with the least monthly family income were more adherent to EBF than those with higher family income. Similar findings have been described by previous studies, which point to the fact that the higher the family income, the less preference toward breastfeeding [11, 20, 37, 38]. This may be explained in part by not having the choice of paying for formula milk and may be less exposed to the adverse effect of formula milk advertising which targets the more privileged mothers.

Limitations in this study include the cross-sectional design, which limits the ability to infer the causation between predictor variables and EBF practice. Using an interview survey may lead to social desirability bias and also recall bias cannot be eliminated. Study participants in our study were completely from the urban population in Hail city, so the generalizability of the result cannot be extended to the rural population in the region. However, the current study provides insight into the rate and factors affecting the adoption of EBF among mothers in the region. Understanding these factors will provide a guide for policymakers and healthcare staff to plan effective breastfeeding health promotion programs to enhance EBF among mothers in the region.

## 5. Conclusion

Our study revealed a relatively higher EBF rate among mothers in Hail region urban community compared to some other regions in Saudi Arabia. Results, also, revealed a number of important modifiable individual and institutional risk factors affecting EBF practice that may be informative when planning for breastfeeding promotion in the region. Adherence to WHO/UNICEF and MOH guidelines for breastfeeding promotion appears to be of value, especially in the private sector.

## Figures and Tables

**Table 1:** Sociodemographic, maternal, child, and health service characteristics reported by the study participants in Hail city, Saudi Arabia, 2019.

Sociodemographic characteristics of the mother	*n*	(%)	95% CI
Mothers' age in years mean ± SD (range)		30.16 ± 7.48 (18–48)
<25	110	24.4	20.6–28.7
25–29	128	28.4	24.4–32.9
30–39	128	28.4	24.4–32.9
40+	84	18.7	15.2–22.6
Mothers' education			
Illiterate	26	5.8	3.9–8.5
Primary	22	4.9	3.2–7.4
Middle	60	13.3	10.4–16.9
Secondary	137	30.4	26.3–35.0
University/higher	205	45.6	40.9–50.3
Mothers' employment status			
Working	135	30.0	25.8–34.5
Housewife	269	59.8	55.1–64.3
Student	46	10.2	7.7–13.5
Family income (SR)			
<3000	16	3.6	2.1–5.8
3000–4999	55	12.2	9.4–15.7
5000–9999	148	32.9	28.6–37.5
10000–14999	169	37.6	33.1–42.2
≥15000	62	13.8	10.8–17.4
Obstetric history and health service-related factors			
Number of pregnancies mean ± SD (range) 3.37 ± 2.12 (1–12)			
<3	67	15.2	12.0–18.9
3-4	260	58.8	54.1–63.4
≥5	115	26.0	22.0–30.4
Number of children under 5 years mean ± SD (range) 1.56 ± 0.62 (1–5)			
≤1	227	50.4	45.7–55.2
≥2	223	49.6	44.8–54.3
Sex of the child			
Male	293	53.1	48.4–57.8
Female	211	46.9	42.2–51.6
Birth order of the child			
First born	107	23.8	20.0–28.0
Second born	106	23.6	19.8–27.8
Third and above	237	52.7	47.9–57.3
No. of antenatal visits mean ± SD (range) 3.97 ± 1.68 (1–9)			
<3	75	16.7	13.4–20.5
3-4	260	57.8	53.1–62.4
5+	115	25.6	21.6–29.9
Breastfeeding counseling received during antenatal care			
Yes	349	77.7	73.5–81.4
No	100	22.3	18.6–26.5
Breastfeeding counseling received immediately after delivery			
Yes	358	79.9	75.8–83.5
No	90	20.1	16.5–24.2
Mode of delivery			
CS	28	6.2	4.3–9.0
Vaginal delivery	421	93.8	91.0–95.7
Place of delivery			
Governmental hospital	402	89.3	86.0–92.0
Private hospital	41	9.1	6.7–12.3
Postnatal follow-up during puerperium			
Yes	280	62.5	57.8–67.0
No	168	37.5	33.0–42.2
Awareness and knowledge about breastfeeding			
Heard about exclusive breastfeeding (yes)	362	80.4	76.4–83.9
Know the meaning of EBF (breastfeeding only)	328	72.9	68.5–76.9
Know the duration of EBF (6 months)	97	21.6	17.9–25.7
What should be done with the colostrum (should be given)	337	74.9	70.6–78.8
Breastfeeding practice of the current child			
Time of initiation of breastfeeding			
During 1^st^ hour of delivery	108	24.0	20.2–28.3
2–24 hours	212	47.1	42.4–51.8
>24 hours	122	27.1	23.1–31.5
Not breastfed at all	8	1.8	0.8–3.6
Place of initiation of breastfeeding			
At the hospital	210	46.8	42.1–51.5
At home	239	53.2	48.5–57.9
Colostrum given to the newborn			
Yes	345	76.8	72.6–80.6
No	104	23.2	19.4–27.4
Prelacteal feeding given to the newborn			
Yes	225	50.1	45.4–54.8
No	224	49.9	45.2–54.6
Practiced exclusive breastfeeding			
Yes	226	50.7	45.9–55.4
No	220	49.3	44.6–54.1

**Table 2:** Bivariate analysis of factors associated with exclusive breastfeeding (EBF) practice among mothers in Hail city, Saudi Arabia, 2019.

Variables	Exclusive breastfeeding		OR (95% CI)	*p* value
	Yes (*n* = 266; 50.7%)	No (*n* = 220; 49.3%)		
Sociodemographic characteristics of the mother				
Mother's age (years)				
<25	71(65.1)	38 (34.9)	1.00 [ref]	0.005
25–29	56 (44.8)	69 (55.2)	0.43 (0.26–0.74)	0.002
30–39	57 (44.5)	71 (55.5)	0.43 (0.25–0.73)	0.002
40+	42 (50.0)	42 (50.0)	0.54 (0.30–0.96)	0.035
Mother's education				
Illiterate	14 (53.8)	12 (46.2)	1 [ref]	0.033
Primary	12 (54.5)	10 (45.5)	1.03 (0.33–3.22)	0.961
Middle	23 (38.3)	37 (61.7)	0.53 (0.21–1.35)	0.185
Secondary	59 (44.0)	75 (56.0)	0.67 (0.29–1.57)	0.360
University/higher	118 (57.8)	86 (42.2)	1.18 (0.52–2.67)	0.698
Mother's employment status				
Working	76 (56.3)	59 (43.7)	1 [ref]	0.005
Housewife	119 (44.9)	146 (55.1)	0.63 (0.42–0.96)	0.032
Student	31 (67.4)	15 (32.6)	1.60 (0.79–3.24)	0.188
Family income (SR)				
<3000	14 (93.3)	1 (6.70)	1 [ref]	
3000–4999	26 (48.1)	28 (51.9)	0.07 (0.01–0.54)	0.011
5000–9999	65 (44.2)	82 (55.8)	0.06 (0.01–0.44)	0.006
10000–14999	89 (53.0)	79 (47.0)	0.08 (0.01–0.63)	0.016
≥15000	32 (51.6)	30 (48.4)	0.08 (0.01–0.62)	0.016
Obstetric history and health service-related factors				
No. of pregnancies				
<3	37 (55.2)	30 (44.8)	1 [ref]	
3-4	139 (53.9)	119 (46.1)	0.95 (0.55–1.63)	0.844
5+	46 (40.7)	67 (59.3)	0.56 (0.30–1.02)	0.060
No. of children under 5 years				
≤1	123 (55.2)	100 (44.8)	1 [ref]	
≥2	103 (46.2)	120 (53.8)	0.70 (0.48–1.01)	0.059
History of breastfeeding the previous child				
Yes	79 (63.7)	113 (40.1)	2.63 (1.70–4.06)	<0.001
No	45 (36.3)	169 (59.9)		
Information about the child under investigation				
Facility where antenatal care received				
Governmental	210 (54.3)	177 (45.7)	3.19 (1.74–5.86)	<0.001
Private	16 (27.1)	43 (72.9)		
No. of antenatal visits				
<3	37 (55.2)	30 (44.8)	1 [ref]	
3-4	139 (53.9)	119 (46.1)	0.95 (0.55–1.63)	0.844
5+	46 (40.7)	67 (59.3)	0.56 (0.30–1.02)	0.060
Breastfeeding counseling received during antenatal care visits				
Yes	184 (53.3)	161 (46.7)	1.58 (1.01–2.48)	0.046
No	42 (42.0)	58 (58.0)	1 [ref]	
Place of delivery				
Governmental hospital	206 (51.8)	192 (48.2)	1.68 (0.87–3.23)	0.120
Private hospital	16 (39.0)	25 (61.0)	1 [ref]	
Mode of delivery				
CS	12 (42.9)	16 (57.1)	1 [ref]	
Normal delivery	213 (51.1)	204 (48.9)	1.39 (0.64–3.02)	0.401
Sex of the child				
Male	118 (50.2)	117 (49.8)	1 [ref]	
Female	108 (51.2)	103 (48.8)	1.04 (0.72–1.51)	0.838
Birth order of the child				
First born	62 (58.5)	44 (41.5)	1 [ref]	
Second born	59 (56.2)	46 (43.8)	0.91 (0.53–1.57)	0.736
Third and above	105 (44.7)	130 (55.3)	0.57 (0.36–0.91)	0.019
Breastfeeding counseling received immediately after delivery				
Yes	199 (56.2)	155 (43.8)	3.34 (2.01–5.54)	<0.001
No	25 (27.8)	56 (72.2)	1 [ref]	
Initiation of breastfeeding				
1^st^ hour	71 (67.0)	35 (33.0)	2.42 (1.53–3.83)	<0.001
>1 hour	155 (45.6)	185 (54.4)	1 [ref]	
Received colostrum				
Yes	204 (59.6)	138 (40.4)	5.51 (3.28–9.25)	<0.001
No	22 (21.2)	82 (78.8)	1 [ref]	
Prelacteal feeding				
Yes	100 (44.8)	123 (55.2)	1 [ref]	0.016
No	125 (56.3)	97 (43.7)	1.59 (1.09–2.30)	
Postnatal follow-up during puerperium				
Yes	149 (62.3)	35 (29.4)	3.97 (2.48–6.38)	<0.001
No	90 (37.7)	84 (70.6)	1 [ref]	
Awareness and knowledge about breastfeeding				
Heard about EBF				
Yes	77 (63.1)	143 (44.1)	2.17 (1.41–3.32)	<0.001
No	45 (36.9)	181 (55.9)	1	
Know the meaning of EBF				
Breastfeeding only	185 (51.5)	174 (48.5)	1.19 (0.75–1.91)	0.460
Other options	41 (47.1)	46 (52.9)	1	
What should be done with the colostrum				
Should be given	193 (57.8)	141 (42.2)	3.28 (2.07–5.20)	<0.001
Should be discarded	33 (29.5)	79 (70.5)	1	

**Table 3:** Multivariate logistic regression model for independent predictors of practice exclusive breastfeeding among mothers in Hail city, Saudi Arabia, 2019.

Variables	aOR	95% CI	*p* value
Education (educated/illiterate)	0.39	0.15–0.99	0.047
Family income (≤3000/>3000 SR)	0.04	0.00–0.31	0.002
Aware about exclusive breastfeeding (yes/no)	3.03	1.78–5.18	<0.001
History of breastfeeding the previous child (yes/no)	2.42	1.46–4.03	<0.001
Facility where antenatal care received (governmental/private)	2.63	1.28–5.41	0.009
Breastfeeding counselling/support received immediately after delivery (yes/no)	2.47	1.34–4.53	0.004
Colostrum feed given (yes/no)	4.24	2.31–7.77	<0.001
Prelacteal feeding given (yes/no)	0.61	0.38–0.97	0.038

Abbreviations: aOR = adjusted odds ratio; SE = standard error; CI = confidence interval; SR = Saudi riyal, equivalent to 0.27 US $. Final −2*∗*log-likelihood: 450.2881; cases included: 402; likelihood ratio: 105.5686; *p* value <0.001.

## Data Availability

The data used to support the findings of this study are available from the corresponding author upon request.

## References

[1] World Health Organization (WHO) (2020). *Breastfeeding Overview*.

[2] Centers for Disease Control and Prevention (CDC) (2020). *Breastfeeding*.

[3] Azuine R. E., Murray J., Alsafi N., Singh G. K. (2015). Exclusive breastfeeding and under-five mortality, 2006-2014: a cross-national analysis of 57 low- and-middle income countries. *International Journal of MCH and AIDS*.

[4] World Health Organization (WHO) (2020). *Indicator Metadata Registry List*.

[5] World Health Organization (WHO) (2020). *Breastfeeding Factsheets. Infant and Young Child Feeding*.

[6] Jones G., Steketee R. W., Black R. E., Bhutta Z. A., Morris S. S. (2003). How many child deaths can we prevent this year?. *The Lancet*.

[7] World Health Organization (WHO)/ (2009). *The United Nations Children’s Fund (UNICEF): Global Action Plan for Prevention and Control of Pneumonia (GAPP)*.

[8] World Health Organization (WHO) (2011). *Regional Office for the Eastern Mediterranean. Regional Strategy on Nutrition 2010–2019/*.

[9] World Health Organization (WHO) (2012). *Resolution WHA65.6. Sixty-Fifth World Health Assembly Geneva*.

[10] El Mouzan M. I., Al Omar A. A., Al Salloum A. A., Al Herbish A. S., Qurachi M. M. (2009). Trends in infant nutrition in Saudi Arabia: compliance with WHO recommendations. *Annals of Saudi Medicine*.

[11] Al Juaid D. A., Binns C. W., Giglia R. C. (2014). Breastfeeding in Saudi Arabia: a review. *International Breastfeeding Journal*.

[12] World Health Organization (WHO) (2020). *Nutrition Landscape Information System, Saudi Arabia Country Profile*.

[13] World Health Organization (WHO) (2017). *Guideline: Protecting, Promoting and Supporting Breastfeeding in Facilities Providing Maternity and Newborn Services*.

[14] World Health Organization (WHO) (2020). *Vaccine-Preventable Diseases: Monitoring System. 2019 Global Summary*.

[15] Mangrio E., Persson K., Bramhagen A.-C. (2018). Sociodemographic, physical, mental and social factors in the cessation of breastfeeding before 6 months: a systematic review. *Scandinavian Journal of Caring Sciences*.

[16] Cernigliaro A., Palmeri S., Immordino P. (2018). Allattamento al seno in Sicilia: analisi della prevalenza, delle disuguaglianze di contesto e di altri fattori di rischio associati (Breastfeeding in Sicily Region (Southern Italy): analysis of prevalence, of contextual inequalities, and of other associated risk factors). *Journal of Preventive Epidemiology*.

[17] Alzaheb R. A. (2017). Review of the factors associated with the timely initiation of breastfeeding and exclusive breastfeeding in the Middle East. *Clinical Medicine Insights Pediatric Journal*.

[18] Behzadifar M., Saki M., Behzadifar M. (2019). Prevalence of exclusive breastfeeding practice in the first six months of life and its determinants in Iran: a systematic review and meta-analysis. *BMC Pediatrics*.

[19] Alzaheb R. A. (2017). Factors influencing exclusive breastfeeding in Tabuk, Saudi Arabia. *Clinical Medicine Insights: Pediatrics*.

[20] El-Gilany A.-H., Shady E., Helal R. (2011). Exclusive breastfeeding in Al-Hassa, Saudi Arabia. *Breastfeeding Medicine*.

[21] Raheel H., Tharkar S. (2018). Why mothers are not exclusively breast feeding their babies till 6 months of age? Knowledge and practices data from two large cities of the Kingdom of Saudi Arabia. *Sudanese Journal of Paediatrics*.

[22] World Health Organization (WHO) (2003). *Global Strategy for Infant and Young Child Feeding*.

[23] Hegazi M. A., Allebdi M., Almohammadi M., Alnafie A., Al-Hazmi L., Alyoubi S. (2019). Factors associated with exclusive breastfeeding in relation to knowledge, attitude and practice of breastfeeding mothers in Rabigh Community, Western Saudi Arabia. *World Journal of Pediatrics*.

[24] Alsulaimani N. (2019). Exclusive breastfeeding among Saudi mothers: exposing the substantial gap between knowledge and practice. *Journal of Family Medicine and Primary Care*.

[25] Victora C. G., Bahl R., Barros A. J. (2016). Lancet Breastfeeding Series Group. Breastfeeding in the 21st century: epidemiology, mechanisms, and lifelong effect. *The Lancet*.

[26] Hunegnaw M. T., Gezie L. D., Teferra A. S. (2017). Exclusive breastfeeding and associated factors among mothers in Gozamin District, Northwest Ethiopia: a community based cross-sectional study. *International Breastfeeding Journal*.

[27] Ochola S. A., Labadarios D., Nduati R. W. (2013). Impact of counselling on exclusive breast-feeding practices in a poor urban setting in Kenya: a randomized controlled trial. *Public Health Nutrition*.

[28] World Health Organization (WHO) (2009). *Baby-Friendly Hospital Initiative: Revised, Updated and Expanded for Integrated Care*.

[29] Karkee R., Lee A. H., Khanal V., Binns C. W. (2014). A community-based prospective cohort study of exclusive breastfeeding in Central Nepal. *BMC Public Health*.

[30] Abdel-Hady D. M., El-Gilany A.-H. (2016). Calculating exclusive breastfeeding rates: comparing dietary “24-hour recall” with recall “since birth” methods. *Breastfeeding Medicine*.

[31] Velusamy K., Premkumar P., Kang G. (2017). Exclusive breastfeeding practices among mothers in urban slum settlements: pooled analysis from three prospective birth cohort studies in South India. *International Breastfeeding Journal*.

[32] Zhao J., Zhao Y., Du M., Binns C. W., Lee A. H. (2017). Maternal education and breastfeeding practices in China: a systematic review and meta-analysis. *Midwifery*.

[33] Vanderlinden K., Van de Putte B. (2017). Pathways of equality through education: impact of gender (in)equality and maternal education on exclusive breastfeeding among natives and migrants in Belgium. *Maternal & Child Nutrition*.

[34] Bonet M., Foix L’Hélias L., Blondel B. (2008). Allaitement maternel exclusif et allaitement partiel en maternité: la situation en France en 2003. *Archives de Pédiatrie*.

[35] Whipps M. D. M. (2017). Education attainment and parity explain the relationship between maternal age and breastfeeding duration in U.S. mothers. *Journal of Human Lactation*.

[36] Chang P.-C., Li S.-F., Yang H.-Y. (2019). Factors associated with cessation of exclusive breastfeeding at 1 and 2 months postpartum in Taiwan. *International Breastfeeding Journal*.

[37] Andrieu N., Goldgar D. E., Easton D. F. (2006). Pregnancies, breast-feeding, and breast cancer risk in the international BRCA1/2 Carrier Cohort Study (IBCCS). *JNCI: Journal of the National Cancer Institute*.

[38] Diab S. S., Alrwely A. K., Elanzy A. A., Elkwikeby A. R., Turky N. (2019). Effects of decline exclusive breastfeeding practice among Saudi adolescent mothers and its risks on the mothers’ and infants’ health at jouf region. *International Journal of Pediatrics and Neonatal Health*.

